# Distribution of essential medicines to primary care institutions in Hubei of China: effects of centralized procurement arrangements

**DOI:** 10.1186/s12913-017-2720-3

**Published:** 2017-11-14

**Authors:** Lianping Yang, Cunrui Huang, Chaojie Liu

**Affiliations:** 1 School of Public Health, Sun Yat-sen University, No.74 Zhongshan Second Road, Guangzhou, Guangdong 510080 China; 2 Global Health Institute, Sun Yat-sen University, Guangzhou, China; 3 School of Health Management, Hubei University of Chinese Medicine, Wuhan, China; 4 School of Psychology and Public Health, La Trobe University, Bundoora, VIC 3086 Australia

**Keywords:** China, Centralized procurement, Distribution, Essential medicine, Primary care

## Abstract

**Background:**

Poor distribution of essential medicines to primary care institutions has attracted criticism since China adopted provincial centralized regional tendering and procurement systems. This study evaluated the impact of new procurement arrangements that limit the number of distributors at the county level in Hubei province, China.

**Methods:**

Procurement ordering and distribution data were collected from four counties that pioneered a new distribution arrangement (commencing September 2012) compared with six counties that continued the existing arrangement over the period from August 2011 to September 2013. The new arrangement allowed primary care institutions and/or suppliers to select a local distributor from a limited panel (from 3 to 5) of government nominated distributors. Difference-in-differences analyses were performed to assess the impact of the new arrangements on delivery and receipt of essential medicines.

**Results:**

Overall, the new distribution arrangement has not improved distribution of essential medicines to primary care institutions. On the contrary, we found a 7.78–19.85 percentage point (*p* < 0.01) decrease in distribution rates to rural primary care institutions. Similar results were demonstrated using the indicator of received rates, with a 7.89–19.65 percentage point (*p* < 0.01) decrease.

**Conclusions:**

Simply limiting the number of distributors does not offer a solution to the poor performance of delivery of essential medicines for primary care institutions, especially those located in rural areas. Procurement arrangements need to consider the special characteristics of rural facilities. In a county, there are more rural primary care institutions than urban ones. On average, rural primary care institutions demand more and are more geographically dispersed compared to their urban counterparts, which may impose increased distribution costs.

**Electronic supplementary material:**

The online version of this article (10.1186/s12913-017-2720-3) contains supplementary material, which is available to authorized users.

## Background

### Health system reform in China

The most recent health system reform in China started in 2009 [1, 2]. The central government was committed to injecting ¥850 billion (US$124 billion) into the health care system over 3 years (from 2010 to 2012), with an aim to [2]: (1) achieve universal (>90%) health insurance coverage; (2) establish a national essential medicines system for primary care; (3) strengthen primary care and public health services delivery; (4) improve equality in access to essential public health services; and (5) test new governance and management models for public hospitals. The reform was designed to lay a solid foundation for affordable and equitable basic health care for all by 2020.

The national essential medicines policy (NEMP) was considered important for containing growth of health expenditure as well as improving safety and quality of primary care. The government recognized that the existing price schedules adopted since 1980s based on a fee-for-service system was distorted. Health institutions were allowed to keep up to 15% of mark-up from sales of medicines, which encouraged over-prescriptions and represented a significant cost driver [3]. In the 1990s, medicine sales accounted for over 50% of revenues in urban health facilities. This figure was even larger in rural health facilities: 70% for outpatient services and 55% for inpatient care [4]. Patients visiting primary care institutions were prescribed on average 2.6 medicines for each encounter - almost one product more than what has been recommended by the World Health Organization (WHO) [3]. Over-prescriptions and irrational use of medicines have since caused serious concerns from the public about the safety and quality of health care [3].

### Changing landscape of supply of essential medicines

China’s economic reforms heavily influenced health system development [1], notably with the introduction of market mechanisms to the health industry since 1978, when China abandoned the decades-long model of a centrally planned economy. A pharmaceutical market rapidly developed when health facilities chose their own medicine suppliers and imposed a 15% profit margin on medicine sales [4–7]. China currently has more than 8000 pharmaceutical manufacturers and 20,000 logistic companies, most of whom have GMP (Good Manufacturing Practice) and/or GSP (Good Supply Practice) accreditations from the National Food and Drug Administration Authority and/or the Bureau of Quality Control [8]. The change from a centrally controlled supply system (push system) to a market-oriented demand system (pull system) [7] improved availability of medicines significantly; however, the benefit of improved availability of medicines has been compromised by medicine price inflation [9]. For reasons of revenue generation, healthcare providers and suppliers preferred highly priced medicines, leaving the hapless consumer to find the money to pay for it [4].

Centralized regional procurement arrangements, first introduced in 2009 as part of the NEMP, have led to dramatic changes in the landscape of medicine provision. Competition in tender processes is intense: many local and small companies have lost to the few giant companies. In some places, “bulk purchasing” turned into “sole purchasing” [10, 11]. In 2010, for example, two companies won rights to deliver essential medicines to 10 out of 11 prefecture-level cities. However, those two companies were not able to cover some local areas because of lack of warehouse and delivery vehicles [10], which suggests a failure of the people conducting the tender process to ensure through due diligence that the bidders could indeed fulfill their contract obligations.

### National Essential Medicines Policy

The NEMP in China contains several components: a National Essential Medicines List (NEML), centralized regional procurement arrangements, and zero-mark-up policy for provision of medicines in primary care facilities [8]. The Ministry of Health (MOH), the National Development and Reform Commission (NDRC) and seven other governmental agencies issued guidelines regarding selection, pricing, supply and distribution, and provision of medicines in primary care facilities [12]. The first NEML compiled by the MOH in August 2009 included 307 generic medicines – 205 western medicines and 102 Traditional Chinese Medicines (TCM) [8]. An updated version published in September 2012 expanded the varieties of essential medicines to 520 (317 western medicines and 203 TCM) [10]. The Chinese NEML was developed based on the WHO model list, considering the needs of local populations, the requirements of primary care facilities, and consumer demands for traditional Chinese medicine. Provincial governments can add additional medicines into their own EML according to their economic situation and specific needs [8]. Government-owned primary care facilities are restricted to only stocking and dispensing medicines listed in EML, all of which must be sold to patients at zero mark-up. Empirical evidence suggests that income from sales of medicines in primary care facilities has fallen and patient charges for medicines have decreased [13–15].

The NEMP aims to improve availability, affordability, and quality use of medicines in primary care facilities [16, 17]. The limited range of medicines defined by the NEML is intended to meet the common needs of consumers and the service capabilities of primary care workers [12]. However, shortages of drug supplies began to attract concerns from the government and the public. Poor distribution of medicines to primary care facilities compounds the difficulties faced by health providers with a limited range of medicines available in the EML. The ability of primary care facilities to provide adequate health care is directly related to the availability of medicines [18].

### Procurement and distribution

The NDRC, which tracks the price and supply of essential medicines in the market, sets price ceilings for medicines on the NEML. Provincial governments established province-based competitive-tenders in each of the 31 provinces/autonomous municipalities, with a goal of reducing prices of medicines while assuring quality. These tenders follow the principles articulated in the “*Opinions on establishing and maintaining a mechanism for good practices in procuring essential medicines for public primary care institutions”* issued by the State Council in November 2010. Primary care facilities must obtain their medicines through suppliers appointed to panel arising from the tender outcomes. The tender process invited pharmaceutical manufacturers and distributors to bid for “price and volume” and “delivery business case” (Two Envelope Selective Tendering System). The outcome of the tender process is an agreed price struck with suppliers, and medicines are therefore procured for the whole province at agreed prices and distributed to primary care centers subject to these collective purchasing arrangements. By doing this, the government is re-gaining control over medicines supply for primary care through full budget support to primary care facilities and the central tendering system.

It is a common practice that several suppliers for each type of medicines (usually with differing prices) are appointed to the supplier panel through the regional tender. For example, five pharmaceutical companies were appointed to the supplier panel for Ampicillin (250 mg) in 2012 for Hubei province. The regional tendering system also publishes a list of distributors (identified through the bidding for business case). In 2015, 305 distributors were included in Hubei, with the majority licensed to distribute medicines within a particular county.

Primary care facilities customarily raise a monthly purchasing order (including supplier-specific types and volumes of medicines) and submit to their regional system through a county platform. The regional system then invites expression of interests from distributors for each type of medicines. Suppliers finally select their preferred distributors (or themselves) to distribute medicines.

### Research question

Poor distribution performance has been blamed for jeopardizing timely supply of medicines to primary care facilities [11]. Because the central government did not include any specific policy on procurement and logistics, regional governments have employed various strategies to ensure supply of medicines in primary care facilities. In Hubei province, the large number of distributors competing in a relatively small market (small orders from primary care institutions, see details in research setting) is suspected to have contributed to poor procurement and logistical performance. Three new models of logistical arrangements have been trialed since September 2012. The first is a medicine-tied distributor – only one distributor is allowed to be selected by a supplier for each type of its listed medicines. The second is a recipient-tied distributor – the primary care facility (recipient) selects one of the four distributors nominated by the government for distributing all of its ordered medicines. The third is a recipient-medicine-tied distributor – a primary care facility selects one of the two distributors nominated by a supplier for each of its listed medicines. As a result, the total number of distributors has fallen dramatically. In those counties that endorsed one of these three new distribution models, the county government selected only 3–5 distributors.

This study aimed to evaluate the three new distribution models. Evidence on effective ways of achieving access and accountability of essential medicines, particularly among low- and middle-income countries, is very limited [19]. The findings of this study will improve our understanding about the complexity of procurement reforms in China.

## Methods

### Settings

We conducted this study in Hubei province. Hubei is located in the middle of China, with a population of 58 million and a GDP of ¥42,686 (US$6892) per capita in 2013 [20].

A total of 416 suppliers and 305 distributors were listed in the Hubei provincial panel of providers responsible for supplying and distributing essential medicines for 1366 primary care facilities across 116 counties (or districts). On average, each county has about 12 primary care facilities (highly urbanized counties may have less primary care facilities and more hospital resources). Although these primary care facilities are geographically dispersed, they all have easy road access.

In Hubei, each primary care facility has about 30 beds, providing an average of 32,073 outpatient consultations and 1044 admissions per year in 2013 (Data source: *2013 Hubei provincial health and family planning development statistics report*).

This study adopted a pre-post comparison design. The intervention group comprised primary care institutions from four counties/districts that pioneered the new distribution arrangements since September 2012. Primary care institutions from six counties with similar socio-economic status were chosen by the Provincial Government to serve as controls.

In the intervention group, two counties implemented the “medicine-tied” distribution model, one implemented the “recipient-tied distributor” model, and another one implemented the “recipient-medicine-tied” distribution model (Table 1).

**Table 1:** Group allocation of primary care institutions and characteristics (2016) of participating counties

Test model	Prefecture	Group	County (or district)	No. of primary care institutions	Population (10,000)	% urban population	Per capita annual income (Yuan RMB)	
							Rural	Urban
Medicine-tied Model								
	Jingmen Municipality	Intervention	Zhongxiang County	29	101.55	56%	16,230	27,940
		Control	Dongbao District	7	37.27	69%	15,980	31,086
			Duodao District	4	30.64	85%	16,420	31,086
			Jingshan County	15	62.73	55%	15,829	27,892
	Yichang Municipality	Intervention	Dangyang County	10	46.86	50%	17,791	29,641
		Control	Yiling District	12	52.41	65%	17,149	30,757
Recipient-tied model								
	Huanggang Municipality	Intervention	Hongan County	11	60.45	41%	9537	23,104
		Control	Tuanfeng County	14	34.26	37%	10,244	22,717
Recipient-medicine-tied model								
	Yichang Municipality	Intervention	Yidu County	11	39.00	60%	17,789	31,195
		Control	Zhijiang County	11	50.15	54%	17,936	28,456

### Interventions

#### Original distribution model (control)

On the 10th day of each month, primary care institutions submit purchasing orders to an online procurement system for approval from their respective county health bureau. The bureau then collates all orders for the entire county and passes the collective order electronically to the head procurement agency at the provincial level.

The provincial procurement agency then develops a monthly purchasing plan and calls for expression of interest from its contracting suppliers (*n* = 416) and distributors (*n* = 305). A supplier (if confirmed by the provincial procurement agency) can choose to distribute medicines by itself, or by one or more qualified distributors endorsed by the provincial procurement agency. A supplier has to disclose its arrangements with the distributors (if it does not distribute the medicines themselves) when it responds to the tender with the provincial procurement agency. Once the order is signed off, the supplier or its delegated distributors are required to deliver the relevant medicines to the locations specified in the order within 72 h.

### New arrangements of distribution model

#### Medicine-tied distributor

Unlike the original system in which a supplier can choose any number of distributors to deliver medicines, the new model restricts the supplier to select from a limited number of distributors (5 in Dangyang and 3 in Zhongxiang) for delivery of medicines within a county. Only one distributor is allowed for each type of medicines. In this distribution model, primary care institutions do not make a choice of distributors.

#### Recipient-tied distributor

In this distribution model, a supplier establishes contracts with 4 distributors selected by the Government for distributing essential medicines. Each primary health institution, as the recipient, is invited to choose one of the selected distributors to deliver all of its ordered medicines.

#### Recipient-medicine-tied distributor

A supplier nominates two distributors for each type of its supplied medicines. The primary care institution chooses one of them as a distributor for the specified medicine it would like to order.

Each pilot county is allowed to select one of the models only (Table 2). Once settled, all primary care institutions in the county have to implement the same model as agreed by the county health bureau.

**Table 2:** Pilot new medicine distribution models and procedures

Distribution Model		Main Procedure
Medicine-tied distributor	Qualified distributors share the task of delivery of medicines, each delivering certain numbers of medicines. Only one distributor is allowed for each type of medicines.	If established contracts between a supplier and the listed distributors exist, they remain valid. Those distributors are eligible to deliver the medicines specified in their contracts. Suppliers can also enter into a new contract with those who do not have an existing contract, for distributing certain types of medicines.
Recipient-tied distributor	One distributor is selected by a primary care institution (from those nominated by suppliers and selected by local government) to deliver all of its ordered medicines.	Existing contracts remain valid between a supplier and the listed distributors. A supplier has to establish a new contract with the listed distributors if no valid contract exists.
Recipient-medicine-tied distributor	A supplier nominates two distributors for each of its supplied medicines. The primary care institution chooses one of them as a distributor for the specified type of medicines.	A supplier nominates two distributors (from the panel list of government approved distributors) for each of its medicines; once accepted, the supplier enters into a contract with the distributors for delivering the specified medicine.

We extracted monthly purchasing order and distribution data for each primary care institution from August 2011 to September 2013 (a total of 26 months). The data set is publicly available on the website (http://www.hbjycg.com/), which contains three items:

- Monetary value (¥) of medicines ordered;
- Monetary value (¥) of medicines delivered;
- Monetary value (¥) of medicines received;

Data from August 2011 to August 2012 were treated as from pre-intervention period, while those from September 2012 to September 2013 were considered as from post-intervention period. However, data from Feb 2013 contained 67% missing values and therefore was excluded from statistical analysis.

### Statistical analysis

Two outcome indicators were calculated based on monthly data:

- Delivery rate (%) = Monetary value (¥) of medicines delivered / Monetary value (¥) of medicines ordered × 100%
- Received rate (%) = Monetary value (¥) of medicines received and in stock / Monetary value (¥) of medicines ordered × 100%

A difference-in-differences (DID) analysis was performed using repeated measures (monthly repetition for each primary health institution) in a mixed regression model (considering the fixed and random effects):

$$ {\mathsf{Y}}_{\mathit{\mathsf{ij}}}={{\mathit{\mathsf{\beta}}}_{\mathsf{1}}}^{\ast }{\mathit{\mathsf{Time}}}_{\mathit{\mathsf{ij}}}+{{\mathit{\mathsf{\beta}}}_{\mathsf{2}}}^{\ast }{\mathit{\mathsf{Intervention}}}_{\mathit{\mathsf{ij}}}+{{\mathit{\mathsf{\beta}}}_{\mathsf{3}}}^{\ast }{{\mathit{\mathsf{Time}}}_{\mathit{\mathsf{ij}}}}^{\ast }{\mathit{\mathsf{Intervention}}}_{\mathit{\mathsf{ij}}}+{{\mathit{\mathsf{\beta}}}_{\mathsf{4}}}^{\ast }{\mathit{\mathsf{Month}}}_{\mathit{\mathsf{ij}}}+{{\mathit{\mathsf{\beta}}}_{\mathsf{5}}}^{\ast }{\mathit{\mathsf{County}}}_{\mathit{\mathsf{ij}}}+{\mathit{\mathsf{\beta}}}_{\mathsf{6}}{\mathit{\mathsf{Size}}}_{\mathit{\mathsf{ij}}}+{\mathit{\mathsf{u}}}_{\mathsf{0}\mathit{\mathsf{j}}}+{\mathit{\mathsf{\varepsilon}}}_{\mathit{\mathsf{ij}}} $$

Y_ij_ is the outcome (dependent) variable: the probability of successful distribution or receipt (in-stock) of medicines. *Time*_*ij*_ indicates the period before (coded as 0) or after (coded as 1) interventions. *Intervention*_*ij*_ indicates the procurement arrangements adopted by each primary care institution (0 = original model, 1 = new model). *Time*_*ij*_ *Intervention_*ij*_ measures the DID effect, with coefficient β_3_ indicating the effect size.

We controlled confounding effects of seasonal changes (dummy variable *Month*_*ij*_), institutional size (*Size*_*ij*_ being categorized into quartiles according to the monetary values of purchasing orders), and county (dummy variable *county*_*ij*_) in the regression models. In the model, Time, Intervention, Time* Intervention, Month, County and Size were introduced with fixed effects; and *institution* was introduced with random effects (u_0j_). We performed the modeling for rural and urban data sets, respectively.

All statistical analyses were performed using Stata (version 12).

## Results

More than 90% of primary care institutions placed a purchasing order every month. Over the 25 month study period, 5% institutions missed 1 month of orders. The rural facilities made larger purchasing orders than their urban counterparts (*p* < 0.01). On average, a monthly order of a rural facility was worth ¥145,929, almost doubled that of an urban facility (¥73,341). (Monetary values (¥) of purchasing orders, delivered and received shown in Additional file 1: Table S1).

Overall, the delivery rates of medicines fluctuated with a slightly declining trend over time. Rural facilities had a lower delivery rate than their urban counterparts (84.0% vs 87.2%, *p* < 0.05).

No significant changes in delivery and received rates were found in the control groups over the periods before and after interventions. However, there was a significant decrease in delivery (from 85.3% to 66.4%, *p* < 0.001) and received (from 84.7% to 66.2%, *p* < 0.001) rates in the rural primary care institutions after they adopted “recipient-tied distribution” model (Table 3).

**Table 3:** Delivery and received rates of medicines in participating institutions before and after interventions

			Delivery rate (%)				Received rate (%)			
			Intervention		Control		Intervention		Control	
Urban			Pre	Post	Pre	Post	Pre	Post	Pre	Post
	Medicine-tied model	No. of orders	65	58	89	80	65	58	89	80
		Mean	97.27	97.68	89.43	83.36	97.27	97.68	89.33	83.19
		SD	3.27	2.78	18.57	27.17	3.27	2.78	18.57	27.18
Rural										
	Medicine-tied model	No. of orders	427	396	429	382	427	396	429	382
		Mean	88.29	84.42	83.27	87.02	88.24	84.25	83.13	86.87
		SD	15.84	21.09	23.67	20.07	15.85	21.10	23.67	20.11
	Recipient-tied model	No. of orders	143	132	169	156	143	132	169	156
		Mean	85.25	66.35	84.08	85.03	84.69	66.15	83.86	84.97
		SD	12.22	28.90	13.59	12.48	12.41	28.83	13.75	12.48
	Recipient-medicine-tied model	No. of orders	130	120	157	132	130	120	157	132
		Mean	78.83	82.10	85.07	84.01	78.82	81.92	84.63	84.00
		SD	22.08	26.17	17.16	21.60	22.08	26.15	17.35	21.60

The DID analyses revealed that the new distribution models did not make a significant difference to urban institutions. However, rural institutions experienced a 7.78 percentage point drop (*p* < 0.01) in delivery rates after they adopted the Medicine-tied distribution model, and a 19.85 percentage point drop (*p* < 0.01) in delivery rates after they adopted the recipient-tied distribution model, respectively (Table 4).

**Table 4:** Effects of new distribution arrangements on delivery rates

Variable	Urban				Rural			
	β	*p*	95%CI		β	*p*	95%CI	
			lower	upper			lower	upper
Medicine-tied model								
Intervention (vs control)	9.63	0.05	0.13	19.12	−12.80	0.00	−18.05	−7.55
Time (pre-post)	−7.01	0.01	−11.96	−2.06	2.94	0.02	0.43	5.45
Intervention*Time	6.32	0.10	−1.15	13.80	**−7.78**	**0.00**	−11.25	−4.31
Recipient-tied model								
Intervention (vs control)	–	–	–	–	−8.89	0.04	−17.20	−0.59
Time (pre-post)	–	–	–	–	1.69	0.34	−1.77	5.14
Intervention*Time	–	–	–	–	**−19.85**	**0.00**	−24.85	−14.84
Recipient-medicine-tied model								
Intervention (vs control)	–	–	–	–	−4.27	0.35	−13.18	4.64
Time (pre-post)	–	–	–	–	−0.76	0.75	−5.41	3.90
Intervention*Time	–	–	–	–	3.99	0.24	−2.67	10.65

*Note*: other variables in the models were not shown in the Table; Bold indicates effect size with statistical significance

Similar findings were demonstrated using the indicator of received rates (Table 5).

**Table 5:** Effects of new distribution arrangements on received rates

Variable	Urban				Rural			
	β	*p*	95%CI		β	*p*	95%CI	
			lower	upper			lower	upper
Medicine-tied model								
Intervention (vs control)	9.66	0.05	0.14	19.19	−13.10	0.00	−18.34	−7.86
Time (pre-post)	−7.08	0.01	−12.02	−2.13	2.93	0.02	0.42	5.43
Intervention*Time	6.40	0.09	−1.06	13.86	**−7.89**	**0.00**	−11.36	−4.43
Recipient-tied model								
Intervention (vs control)	–	–	–	–	−9.17	0.03	−17.50	−0.84
Time (pre-post)	–	–	–	–	1.83	0.30	−1.63	5.29
Intervention*Time	–	–	–	–	**−19.65**	**0.00**	−24.67	−14.63
Recipient-medicine-tied model								
Intervention (vs control)	–	–	–	–	−3.68	0.42	−12.57	5.22
Time (pre-post)	–	–	–	–	−0.26	0.91	−4.93	4.41
Intervention*Time	–	–	–	–	3.37	0.32	−3.31	10.05

*Note*: other variables in the models were not shown in the Table; Bold indicates effect size with statistical significance

## Discussion

### Key findings

These three new medicine distribution strategies fail to achieve their goals, despite great efforts made by the government. Over the past decades, a competitive medicine distribution market has developed in China; however, the market has heavily biased towards highly profitable and expensive medicines [3, 4, 9]. The assumption behind the new distribution arrangements is that by reducing the number of contenders, distributors are more likely to be attracted to delivery of essential (cheap) medicines because of economy of scale. Unfortunately, evidence generated from this study shows that it is not the case. None of the new distribution models demonstrated improvement in delivery and received rates. This finding is consistent with studies undertaken elsewhere in China [10, 11, 21]. Yao and Xiao [11] found that governmental interventions often favor large distributors and those large distributors may overlook primary care market.

The negative impact of the new distribution strategies on rural primary care facilities is concerning. We found that the “one distributor for one medicine” policy is associated with a drop of 7.78 percentage points in delivery rates for rural facilities. The “one distributor for one facility” policy led to even greater decrease in delivery rates: 19.85 percentage points. Given the relatively insignificant impact of the new arrangements on urban facilities, the urban-rural disparity in relation to supply of essential medicines actually enlarged. This study revealed that rural primary care facilities tend to place larger purchasing orders than their urban counterparts do. This may be a reflection of general shortage of health resources in rural areas, or stockpiling in anticipation of failure to deliver. Urban consumers can readily seek alternative services from other providers given the highly concentrated nature of resource distribution in urban areas, but rural consumers have fewer options. Consequently, rural primary care facilities have to store more medicines than their urban counterparts. Low distribution rates of essential medicines are likely to impose a disproportional impact on rural consumers.

Our findings showed that simply limiting the number of distributors does not offer a solution to the poor performance of delivery of essential medicines for primary care institutions, especially those located in rural areas.

A potential monopoly in medicine distribution may have several consequences. Shortage of medicines supply occurred after local small distributors, who used to supply medicines to primary care facilities, lost their rights to large competitors in the centralized tendering system. Studies showed that only one or two distribution companies have the ability to deliver medicines to every primary care facility in a region [11]. Even if they win the tendering, rural primary care facilities are likely to shoulder disproportional consequences of delay and shortage in supply of medicines simply because of their geographically dispersed locations and high delivery costs. Meanwhile, due to the cumbersome arrangements as described above, the majority of primary care facilities are forced to change medicines distributors, leading to increased administrative and transaction costs.

### Policy implications

Lessons from China may have implications to the international community. Shortage of supply of essential medicines is a critical health systems issue worldwide, especially for middle- and low-income countries where health resources are typically limited. Findings of this study are particularly relevant to the countries where NEMP is regarded as an effective measure for ensuring accessible, affordable and equitable healthcare services. Most of those countries face quite similar challenges: inequitable urban concentration of resources; higher profit margins for more expensive medicines; lack of or inefficient operations of public supply chain for essential medicines; and low management capacity of primary care institutions [22].

Previous studies showed that there is no consensus on whether a decentralized or centralized system can enhance supply chain and improve supply of medicines [23, 24]. A centralized system is thought to consolidate purchasing power, bringing benefits of reduced prices and transaction costs, improved quality assurance, reduced procurement corruption, standardization, and increased equity [25]. But experiences from Ghana and Guatemala [24] suggest that logistics systems can be effectively decentralized for some functions while others should remain centralized. Centralization is associated with better performance in inventory control and information systems; whereas decentralization is associated with better performance in planning and budgeting. Higher logistics decision space at the local level produced higher procurement performance in Guatemala (where an open contracting supplier system was adopted), but lower procurement performance in Ghana (where medicines were purchased from public warehouses). In Guatemala, medicines were more likely to be supplied in full and on time when local health facilities were allowed to fill in medicines outside of the contracting system. In Ghana, those who were allowed to make their own procurement decisions were more likely to purchase medicines off the NEML from private suppliers [24]. Tanzanian recently changed its medicines delivery system from a central ‘push’ system to a decentralized ‘pull’ system. Instead of providing pre-packaged “kits” of essential medicines through central control, local primary care facilities were asked to order individual medicines. Unfortunately, the ‘pull’ system failed to adequately address accountability concerns arising from the ‘push’ system, which was blamed for leading to frequent stock-out of medicines [18]. Poor record keeping, lack of capacity of local facility staff to correctly order and manage medicines, and changing clinical treatment guidelines may have compromised the effects of decentralized procurement arrangements [18].

In most health systems, the pharmaceutical supply chain tends to let market determine resource allocation. Liu argued that a fine balance between market competition and governmental intervention is needed [26]. Too many small competitors are detrimental to economy of scale; whereas too few may lead to concentration of market power and neglect of low end (less profitable) market. Ebenezer Tetteh calls for a private distribution system consisting of a consolidated wholesale business and multiplicity of retail pharmacies for African countries [22]. Tetteh argued that a public supply chain is still needed when the private sector is involved in distributing essential medicines. The public supply chain can re-direct efforts and resources to improving medicines delivery to rural settings, which are likely to be ignored by the private sector. The public supply chain may also be shortened through support from private distributors and retail pharmacies [22].

It is important to note that the Chinese system is unique in many aspects. While China may be able to claim universal coverage of essential medicines through its strong governmental control over provision of essential medicines, the poor distribution of essential medicines especially for rural primary care facilities deserves increasing attention. The majority of pharmaceutical distributors in China are private, and the market force can bring disadvantages to rural health facilities due to high costs of distribution. It is a great challenge to ensure accountability of private suppliers and distributors of medicines.

### Limitations

This study drew upon a natural experiment, in which participating primary care facilities were not randomly selected and allocated. We were not able to consider variances among the participating facilities in terms of scope of services and consumer demands due to unavailability of data. However, participating facilities in the control group operate in a similar socio-economic environment compared with those in the experimental group.

This study captured only short-term (over a year) effects of the new procurement arrangements. Further studies are needed to investigate the long-term outcome of the new arrangements.

## Conclusions

The Chinese government has made sincere efforts to improve accountability of the essential medicines supply system. It may be unfair to blame the centralized regional procurement arrangements for the shortage of supply of essential medicines in primary care facilities; however, government intervention may not necessarily be able to rectify “market failure” without consequences. Concentration of market power by means other than fair competition in medicines logistics is likely to disadvantage smaller rural primary care facilities, enlarging urban-rural disparities in supply of essential medicines. We posit that governmental enforcement of NEML and procurement standards is a reasonable step towards a more transparent and accountable system. However, the government should also consider in detail the range of logistics functions that decision powers can be delegated to local health facilities. A more flexible selection process involving more small local distributors may bring benefits to primary care facilities, especially those located in rural areas. By doing so, local health facilities are given more “decision space”, which may be able to mitigate some of the unintended consequences (i.e. enlarged urban-rural gap in supply of medicines) associated with centralized procurement [24]. However, it is important to ensure that decentralized arrangements are aligned with the capacities of lower level of administrations.

## Additional file

Additional file 1: Table S1. Monetary values (¥) of purchasing orders, delivered and received. Description of data: details of all orders, delivered amount, received amount of essential medicines. (DOCX 16 kb)

## Abbreviations

DID: Difference-in-differences
MOH: Ministry of Health
NDRC: National Development and Reform Commission
NEML: National Essential Medicines List
NEMP: National essential medicines policy
TCM: Traditional Chinese Medicines
