# Supplementary material for: Distribution of essential medicines to primary care institutions in Hubei of China: effects of centralized procurement arrangements
Source: BMC Health Serv Res. 2017 Nov 14;17:727. doi: 10.1186/s12913-017-2720-3 (PMC5686827; doi:10.1186/s12913-017-2720-3)
Supplement: Additional file 1: Table S1. — Monetary values (¥) of purchasing orders, delivered and received. Description of data: details of all orders, delivered amount, received amount of essential medicines. (DOCX 16 kb) [file 12913_2017_2720_MOESM1_ESM.docx]

**Supplementary materials**

**Table S1 Monetary values (**¥**) of purchasing orders, delivered and received**

|  |  |  | **Ordered** | | | | **Delivered** | | | | **Received** | | | |
| --- | --- | --- | --- | --- | --- | --- | --- | --- | --- | --- | --- | --- | --- | --- |
|  | **Distribution model** | Variables | **Intervention** | | **Control** | | **Intervention** | | **Control** | | **Intervention** | | **Control** | |
|  |  |  | **Pre** | **Post** | **Pre** | **Post** | **Pre** | **Post** | **Pre** | **Post** | **Pre** | **Post** | **Pre** | **Post** |
| **Urban** |  |  |  |  |  |  |  |  |  |  |  |  |  |  |
|  | **Medicine-Unique model** | No. orders | 65 | 58 | 89 | 80 | 65 | 58 | 89 | 80 | 65 | 58 | 89 | 80 |
|  |  | Mean | 45134.2 | 46678.6 | 84667.7 | 118366.5 | 44248.9 | 45849.5 | 75162.3 | 104175.8 | 44248.9 | 45849.5 | 75106.1 | 103808.8 |
|  |  | SD | 28505.3 | 23791.8 | 91180.0 | 112922.9 | 28559.7 | 23902.5 | 86996.3 | 101469.9 | 28559.7 | 23902.5 | 86995.0 | 101155.5 |
|  |  | Median | 38625.6 | 39980.4 | 44440.1 | 70226.1 | 37242.5 | 39090.9 | 40382.0 | 56429.0 | 37242.5 | 39090.9 | 40382.0 | 56365.1 |
|  | **Recipient-tied model** | No. orders | - | - | 20 | 23 | - | - | 20 | 23 | - | - | 20 | 23 |
|  |  | Mean | - | - | 3695.3 | 7740.3 | - | - | 3076.8 | 5293.3 | - | - | 3076.8 | 5293.3 |
|  |  | SD | - | - | 4672.6 | 5266.4 | - | - | 4470.1 | 5294.1 | - | - | 4470.1 | 5294.1 |
|  |  | Median | - | - | 1341.2 | 5328.1 | - | - | 832.8 | 3541.3 | - | - | 832.8 | 3541.3 |
|  | **Recipient-medicine-tied model** | No. orders | 13 | 12 | - | - | 13 | 12 | - | - | 13 | 12 | - | - |
|  |  | Mean | 122210.3 | 164193.2 | - | - | 110235.7 | 146579.7 | - | - | 110052.4 | 146579.7 | - | - |
|  |  | SD | 31698.0 | 96734.1 | - | - | 27366.0 | 101000.2 | - | - | 27362.6 | 101000.2 | - | - |
|  |  | Median | 118366.5 | 129115.9 | - | - | 112621.8 | 109545.4 | - | - | 110370.2 | 109545.4 | - | - |
| **Rural** |  |  |  |  |  |  |  |  |  |  |  |  |  |  |
|  | **Medicine-Unique model** | No. orders | 427 | 396 | 429 | 382 | 427 | 396 | 429 | 382 | 427 | 396 | 429 | 382 |
|  |  | Mean | 154771.1 | 184903.5 | 124183.1 | 171005.3 | 136046.5 | 157467.8 | 99900.5 | 150216.8 | 135966.6 | 157171.0 | 99667.0 | 149970.2 |
|  |  | SD | 105853.0 | 137821.7 | 86161.5 | 87394.7 | 99351.5 | 130888.4 | 74082.5 | 84995.8 | 99330.8 | 130799.3 | 73852.9 | 85026.9 |
|  |  | Median | 128945.5 | 147306.8 | 100752.6 | 156071.5 | 107605.4 | 121110.3 | 79700.0 | 141407.6 | 107605.4 | 121041.6 | 79700.0 | 141234.1 |
|  | **Recipient-tied model** | No. orders | 143 | 132 | 169 | 156 | 143 | 132 | 169 | 156 | 143 | 132 | 169 | 156 |
|  |  | Mean | 174903.2 | 217892.6 | 62663.2 | 86970.3 | 143671.6 | 149216.0 | 52156.1 | 74977.6 | 142063.2 | 148767.4 | 52043.7 | 74901.0 |
|  |  | SD | 92006.6 | 94497.5 | 42478.7 | 45641.4 | 65552.6 | 89428.6 | 35915.9 | 41298.3 | 62386.4 | 89195.8 | 35938.0 | 41202.5 |
|  |  | Median | 162602.3 | 211370.9 | 54169.4 | 87401.7 | 142335.1 | 152945.4 | 46503.8 | 75264.5 | 141157.0 | 152649.2 | 46503.8 | 75264.5 |
|  | **Recipient-medicine-tied model** | No. orders | 130 | 120 | 157 | 132 | 130 | 120 | 157 | 132 | 130 | 120 | 157 | 132 |
|  |  | Mean | 57479.9 | 113499.0 | 172244.4 | 198389.1 | 45196.8 | 90132.9 | 148433.4 | 168952.9 | 45192.6 | 89912.8 | 147878.3 | 168939.3 |
|  |  | SD | 41795.7 | 77275.0 | 132784.3 | 129664.0 | 38198.4 | 73366.4 | 123736.6 | 124666.5 | 38187.8 | 73259.0 | 123533.9 | 124660.4 |
|  |  | Median | 44900.3 | 103379.1 | 150110.2 | 187428.2 | 32020.2 | 71907.4 | 120095.6 | 150260.7 | 32020.2 | 71907.4 | 119240.4 | 150260.7 |
